# Neurophysiological characteristics of reward processing in deaf college students under different social contexts

**DOI:** 10.3389/fnins.2025.1524443

**Published:** 2025-03-10

**Authors:** Xue Du, Ting Huang, Shiqiong Wu, Xingru Wang, Xiaoyi Chen

**Affiliations:** Key Laboratory of Applied Psychology,Chongqing Normal University, Chongqing, China

**Keywords:** deaf college students, reward processing, social context, ERPs, neurophysiological characteristics

## Abstract

**Background:**

In the context of social exclusion, individuals tend to make choices that are advantageous to themselves and optimize their interests. Due to hearing impairment, deaf college students face more social exclusion in our society. However, the neural mechanisms of reward processing in deaf college students during different situations of social exclusion remain unknown.

**Methods:**

A total of 27 deaf college students completed the monetary and social reward delay tasks while recording event-related potential (ERP) data.

**Results:**

The behavioral hit rate was sensitive to the main effect of social context; that is, the deaf college students showed a higher hit rate in social inclusion than in social exclusion. The amplitude of Cue P3 elicited by reward cues was found to be higher in social exclusion than in social inclusion, particularly when the amplitudes of monetary cues were higher than those of social cues. In the reward feedback outcome phase, small magnitude induced a greater feedback-evoked P3 than large magnitude. Additionally, they exhibited a large feedback-related negativity amplitude for large-magnitude (but not for small-magnitude) monetary reward cues.

**Conclusion:**

Deaf college students were more sensitive to reward cues in social exclusion than in social inclusion, especially to monetary cues, and more concerned with attaining greater monetary gains.

## 1 Introduction

Human beings are shaped by their social relationships that create their environment and influence mental health (Yoshioka and Noguchi, 2009). These influences are impacted by the nature of the environment and can have different outcomes, such as social inclusion, acceptance, or support, that have been widely observed to be associated with pleasant feelings, healthy physiological functions, and good physical health (Eisenberger, 2013; Eisenberger et al., 2003; Kiecolt-Glaser et al., 2010; Pressman et al., 2019). Social exclusion or rejection induces negative or painful emotions (Jones et al., 2011; Wesselmann et al., 2022), which make individuals more inclined toward self-interest when faced with decisions or choices (Li et al., 2010). For example, individuals who have experienced rejection may exhibit heightened fear of negative social evaluation (Twenge et al., 2007) and are too sensitive to monetary reward cues (Xu and Wang, 2021; Xu et al., 2022). In summary, social exclusion has an effect on reward sensitivity in college students (Groschwitz et al., 2016; Olié et al., 2017). Due to their hearing impairments, deaf college students might isolate themselves in a passively exclusionary environment, akin to other excluded groups in many communication processes, owing to a mismatch of modalities and information (Spisak and Indurkhya, 2023). Students who are accustomed to using spoken language can receive and convey more information of higher quality, whereas deaf students who have grown up using sign language education often have difficulty communicating with hearing college students (Stinson et al., 1996). Therefore, it is important to examine the characteristics of reward processing in deaf college students in different social contexts.

The reward process is an effective method for influencing behavior and reflecting individuals’ motivations, expectations, and other mental processes. It includes two components: expectations and feedback on results (Maunsell, 2004; Wesselmann et al., 2022). The monetary incentive delay (MID) task represents a classic paradigm commonly used in reward processing research (Balodis and Potenza, 2015; Gu et al., 2019; Wilson et al., 2018) that measures reward-related expectancy, attention, and motivational mental processes (Zhang et al., 2020). The social incentive delay (SID) task was developed based on the monetary incentive delay task by using emotional faces instead of money as reward feedback materials (Smoski et al., 2011). Previous studies have indicated that hearing college students prioritize money over social rewards (Altikulaç et al., 2019; Rademacher et al., 2010). It is well established that both monetary incentives (Cristofori et al., 2015) and social rewards (such as social support) (Morese et al., 2019) can mitigate the negative consequences of social exclusion. Studies have explored the regulatory effect of social exclusion on reward sensitivity and found that social exclusion can increase an individual’s sensitivity to rewards (Chen et al., 2017). For instance, social exclusion has been demonstrated to increase an individual’s desire for monetary rewards (Zhou and Gao, 2008). Conversely, acute stress has been shown to reduce the degree of striatal activation in response to monetary rewards, suggesting that social exclusion may result in a blunted brain response to these rewards (Ossewaarde et al., 2011). In addition, individuals who have experienced rejection showed higher sensitivity to social rewards compared to monetary rewards (Mead et al., 2011). Considering these differing perspectives, the present study investigated the neurophysiological characteristics of reward processing mechanisms in deaf college students across diverse social contexts by using the MID and SID tasks.

The current study depended on the event-related potential (ERP) technique since its high temporal resolution enables recognizing substages within both anticipatory and consummatory reward processing mechanisms (Ait Oumeziane et al., 2019), enabling a direct comparison of social and monetary reward processing (Greimel et al., 2018). This study focused on two ERP components to investigate anticipatory and consummatory reward processing, the significance of which has been confirmed by previous studies using both the MID and SID tasks (Ait Oumeziane et al., 2017; Flores et al., 2015).

The P3 and feedback-related negativity (FRN) are components of the electroencephalogram (EEG) that are often associated with the anticipation and feedback phases of reward (Zhang et al., 2020). In previous studies, P3 has been found to be a positive deflection and is considered one of the reward-related electrophysiological markers (Broyd et al., 2012; Pfabigan et al., 2014; Vignapiano et al., 2016). The amplitude of P3 is related to the individual’s attention allocation and motivation for the task and the value of the stimulus (Groom et al., 2009; Polich and Kok, 1995; Vignapiano et al., 2016). Additionally, it has been shown that P3 associated with the expectation of monetary rewards, the Cue P3 response has greater amplitude for anticipatory tasks that represent the ability to obtain monetary cues (Pfabigan et al., 2014). Furthermore, P3 amplitude is greater for monetary reward cues than for neutral cues. Studies examining other types of rewards, including monetary rewards, have shown that P3 has a greater amplitude for larger rewards (Goldstein et al., 2006). Combined with the above-mentionedand the behavioural performance of individuals inthe context of social exclusion (Feng et al., 2021), we estimatedthat deaf college students may be more inclined toward monetary reward cues than neutral cues.

FRN is a negative component associated with feedback results (Li et al., 2018), mainly induced when results are evaluated (Yuan et al., 2012). The FRN reaches its maximum approximately 200–300 ms after the onset of the outcome feedback, exhibiting a more negative deflection for unfavorable outcomes (e.g., monetary losses) compared to favorable feedback (Gehring and Willoughby, 2002; Glazer et al., 2018; Holroyd and Coles, 2002; Miltner et al., 1997). This reflects an initial binary evaluation of outcomes as better or worse than expected (Foti et al., 2011; Holroyd et al., 2008). Source localization suggests that the intracerebral source of FRN occurrence is in the vicinity of the anterior cingulate cortex (ACC), which is associated with behavioral decision-making and cognitive control. Consequently, the FRN component reflects an individual’s learning and decision-making processes in the context of outcome evaluation (Holroyd and Coles, 2002; Holroyd and Yeung, 2012; Li et al., 2018; Xiang et al., 2008). This study, therefore, explored whether the deaf college students exhibit comparable reactions to rewarded feedback during this stage and the distinctions in feedback across different reward categories.

This study aimed to explore the behavioral responses and the corresponding ERP responses of deaf college students’ reward processing in the context of social exclusion and inclusion. This may facilitate a further understanding of the characteristics of deaf college students from the perspective of reward and provide preliminary suggestions for their future life and studies.

## 2 Methods

### 2.1 Participants

We used G*Power 3.1 for estimating the sample size (Faul et al., 2009). The minimum sample size required for this study was 23 to achieve a test power of 0.95 (*α* = 0.05) at a medium effect size (0.25). A total of 27 deaf college students from Chongqing Normal University were recruited to participate in this experiment (16 women, mean age: 20.44 years), matched by age and gender. All the deaf participants were college students enrolled in the Department of Special Education of the Normal University. They entered the university through a single examination and a single enrollment for undergraduate education, had access to the same educational resources as ordinary college students, and were also eligible for master’s degree programs. The severity of hearing loss was categorized based on the audiological records as moderate (40–69 dB hearing level), severe (70–94 dB hearing level), or profound (≥95 dB hearing level) (Stevenson et al., 2011). All deaf college students were proficient in sign language, and their hearing impairment was defined at the time of diagnosis by an otorhinolaryngologist or audiologist (Dyck et al., 2004). It was verbally reported that they had an average hearing loss of 71 dB or more in both ears. Except for hearing impairment, the participants had no history of mental illness, had normal or corrected vision, were not color blind, had not participated in similar experimental studies before, and did not know the purpose of the experiment. Each participant signed the informed consent form prior to the experiment and voluntarily participated after understanding the risks and benefits involved. The research was approved by the local ethics committee (Institute of Psychology, Chongqing Normal University).

After all experiments were completed, the participants were asked to complete the following questionnaires: (1) the Sensitivity to Reward Questionnaire (SRQ) (Torrubia et al., 2001), which measures reward sensitivity, with a high score indicating a high level of sensitivity to reward (range = 0–24); (2) the Chinese version of State–Trait Anxiety Inventory Form Y (STAI-Y) (Cheng et al., 2021), which measures state–trait anxiety, with a high score indicating a high level of anxiety (range = 20–80); and (3) the Chinese version of the Beck Depression Inventory-II (BDI-II-C) (Yang et al., 2014), with a high score indicating a high level of depressive tendency (range = 0–63). The questionnaires were provided in written form and explained to the participants. Table 1 presents the demographic and clinical characteristics of participants.

**Table 1:** Demographic and clinical characteristics of participants (*N* = 57).

Measure	Hearing students	Deaf students	Between groups *p*-Value
Participants	30	27	
Sex (male/female)	15/15	11/16	0.325
Age (years)	20.30 (1.97)	20.44 (1.60)	0.764
SRQ	11.18 (3.33)	10.61 (3.61)	0.535
TAI	45.85 (4.26)	45.34 (6.50)	0.717
SAI	37.26 (10.33)	43.35 (9.44)	0.022
BDI-II	7.21 (7.51)	13.77 (8.54)	0.003

SRQ, the Sensitivity to Reward Questionnaire; TAI, SAI, the Chinese version of State–Trait Anxiety Inventory Form Y; BDI-II, the Beck Depression Inventory, second edition. *p < 0.05, **p < 0.01, and ***p < 0.001.

There were no significant differences in reward sensitivity or trait anxiety between hearing and deaf college students (all *p* > 0.05). However, significant differences were found in state anxiety and depression, with deaf college students exhibiting higher levels of anxiety and depression compared to their hearing counterparts.

### 2.2 Stimuli

Social context pictures (30 each of exclusion/inclusion context) were selected from the image database of social inclusion and exclusion in young Asian adults (ISIEA) (Zheng et al., 2021). The different reward cues and types of feedback were mainly based on the classic paradigm of reward processing (MID). The monetary reward feedback was a clear picture of ¥1 Chinese yuan (approximately US$0.15) and a clear picture of ¥0.1 (approximately $0.015). The social reward feedback was a picture of a smiling expression only, a neutral expression only, and a picture of a smiling expression and a thumbs-up with both hands given by volunteers (who were not known to any of the subjects participating in the experiment).

### 2.3 Experimental design and procedure

The procedure was programmed and performed using E-prime 3.0. The study use d high-temporal resolution electroencephalogram (EEG) techniques to identify responses during the reward processing expectation and outcome evaluation phases. Before the start of the formal experiment, participants were asked to perform a practice experiment to familiarize themselves with the procedure and understand the difficulty of the experimental task and were allowed to start the experiment only when they met the criteria.

**Figure 1:**
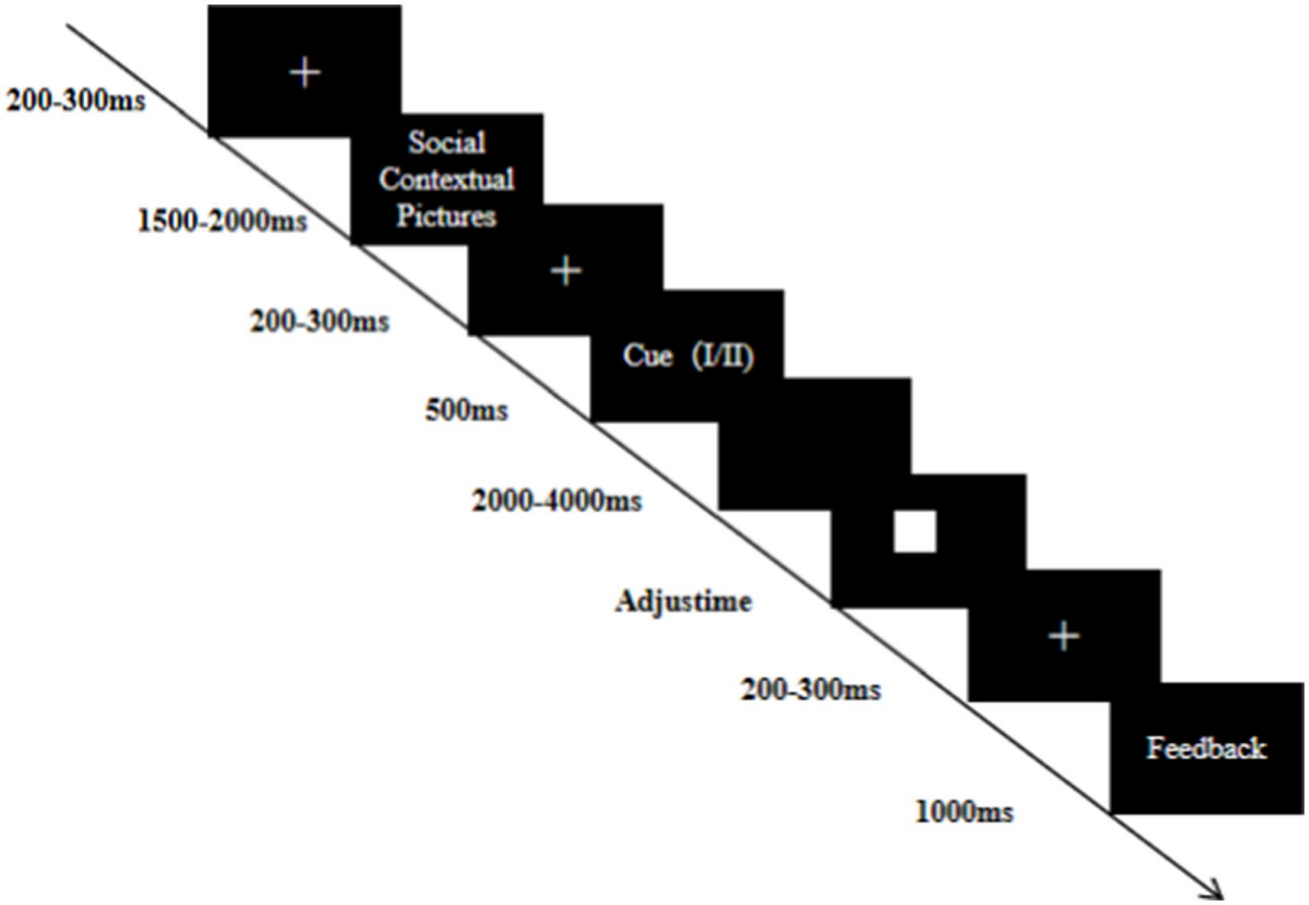
Example of a trial flow for the research experiment.

The formal task consisted of two MID and two SID blocks. In both the MID and SID blocks, each trial began with a fixation cross that appeared at the center of the screen for 200–300 ms. A picture of the social context (exclusion or inclusion) would be presented for 1,500–2000 ms, and the participants need only to carefully observe the picture of the experience situation and complete the later task with the feelings experienced. Then, the reward magnitude prompt cue I (small magnitude) /II (large magnitude) appeared, indicating the amount of potential reward for 500 ms, followed by a blank screen for a random duration ranging from 2000 to 4,000 ms. Subsequently, a white square would appear at the center of the screen, and the participants were required to press the “Q” key as quickly as possible to gain a reward. Finally, the center of the screen presented feedback on the results of the white square. If participants’ reaction time was faster than the duration of the presentation of the white square (target), the ongoing trial would be considered as a ‘hit’ trial. Conversely, it would be considered a ‘miss’ trial. After responding to the target, each participant received monetary or social feedback for 1,000 ms. Each MID or SID block consisted of 40 small-reward trials and 40 large-reward trials and a total of 320 trials. The experimental trial flow of this study is shown in Figure 1.

Regarding the MID blocks, participants received a picture of ¥0.1 Chinese yuan (approximately US$0.015) after successfully hitting the target but a scrambled picture of ¥0.1 (indicating no monetary gain) after missing that target in each trial of the small-reward condition; meanwhile, the feedback was a picture (or scrambled picture) of ¥1 (approximately $0.15) in the large-reward condition. Regarding the SID blocks, participants received a picture showing a person with a smiling face after they successfully hit the target but a person with a neutral facial expression after they missed that target in each trial of the small-reward condition; meanwhile in the large-reward condition, feedback for hits was a picture showing a person who smiled and gave a thumbs up, while feedback for missed was a person with neutral facial expression.

**Figure 2:**
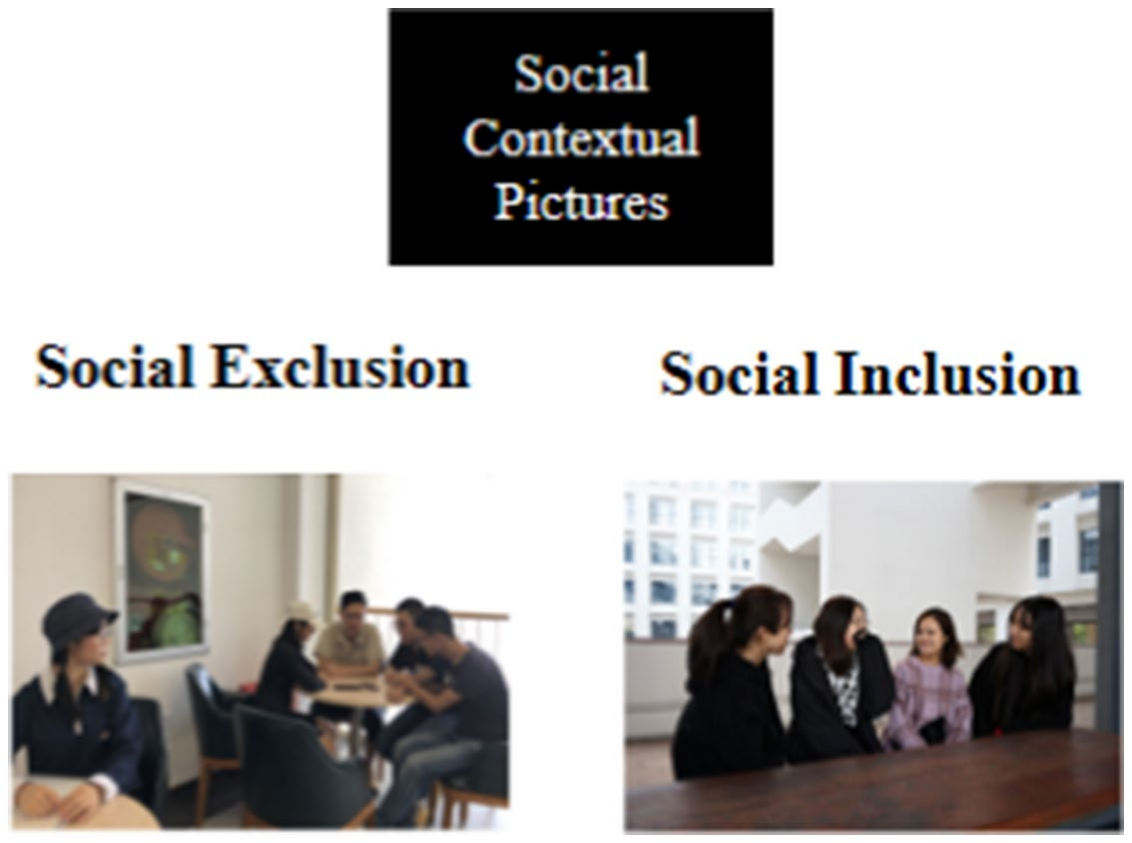
Examples of social contextual pictures.

**Figure 3:**
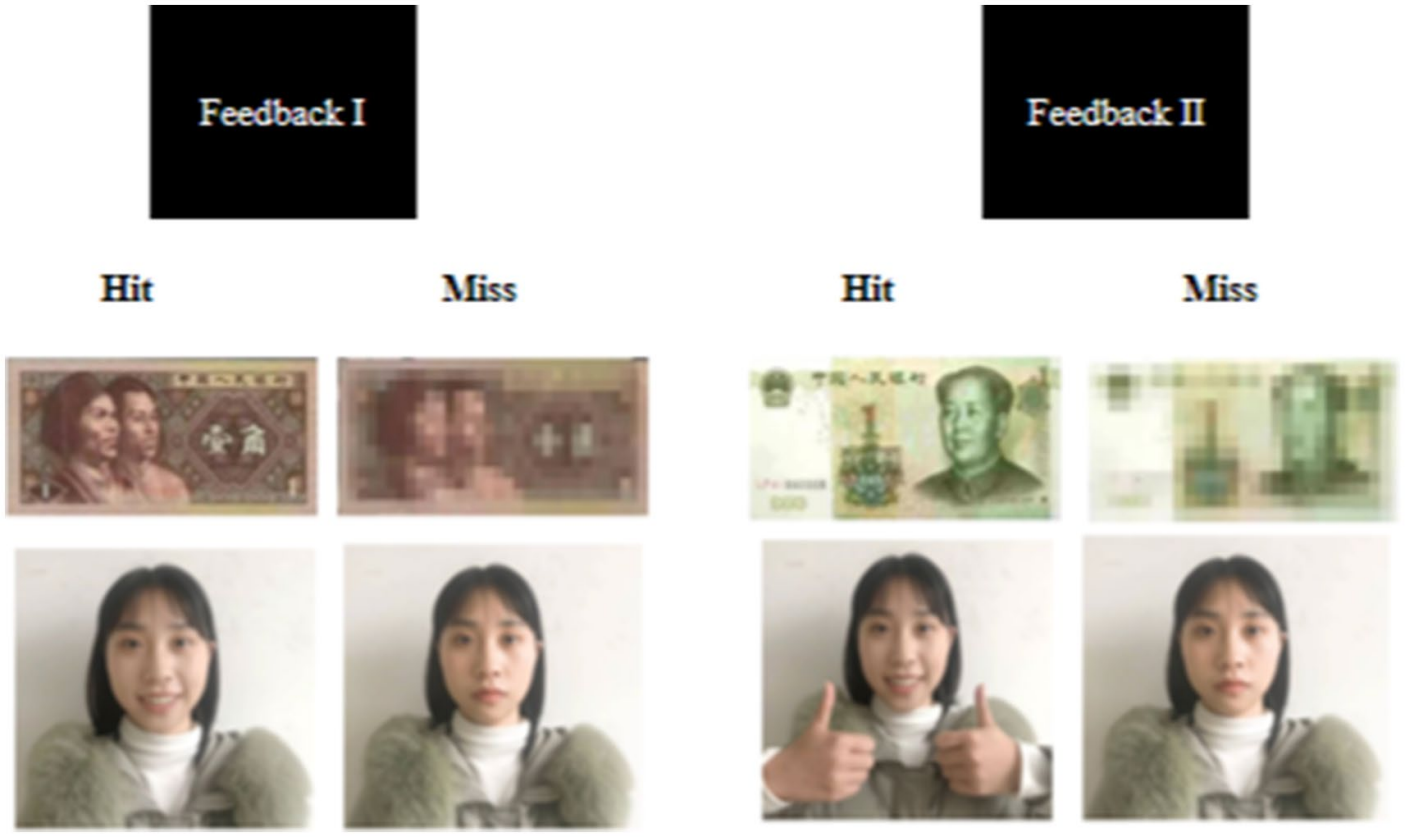
Monetary delayed reward task and social delayed reward task hit and miss feedback images, respectively.

Thirty situation images of social exclusion and social inclusion were selected from each after screening in the gallery, and social exclusion/inclusion images were randomly presented in each section. The SID task provided socially relevant information (e.g., friendly faces) rather than monetary feedback (Rademacher et al., 2010; Spreckelmeyer et al., 2009), and in addition, pictures of women’s faces were used for the experiment because they are more relatable and activate subjects’ reward circuitry than pictures of men (Aharon et al., 2001). Images of task expressions and movements in the SID section were captured by a student volunteer at the school and then informed that the images were only used in the experimental study and the participants did not know them before obtaining consent for use in the experiment. The picture examples of social exclusion and social inclusion situations are shown in Figure 2, and the feedback information is shown in Figure 3.

### 2.4 EEG recording and analysis

The EEG recording and subsystem of Brain Products in Germany and the 64-lead electrode cap were used to collect EEG data. The preprocessing and analysis of EEG data were performed using MATLAB R2016a (Math Works, USA) and EEGLAB 13.6.5b components. Based on previous studies conducted on deaf individuals (Dong et al., 2024; Gong et al., 2022; Sun et al., 2024), bilateral mastoids were used as reference electrodes (bilateral mastoids were averaged for reference), the lateral electrodes of both eyes recorded horizontal electro-oculography electricity (HEOG), and the upper and lower electrodes of the left eye recorded vertical electro-oculography electricity (VEQG). The scalp chainsaw at each electrode is kept below 5 kΩ. The EEG signal was filtered in a range of 0.05 ~ 100 Hz and sampled at the rate of 500 Hz. After completing continuous EEG recording, the data were processed offline, and the offline analysis period was 1,200 ms, including 200 ms for feedback stimulus presentation money (as a baseline) and 1,000 ms for analysis after presentation. Trials with severe electromyogram (EMG) interference were excluded, and eye movement artifacts were corrected by independent component analysis (ICA) algorithm (Jung et al., 2001).

According to the purpose of this study, the main focus is on the characteristics of reward expectation and feedback of deaf college students in different reward categories and magnitudes under different social contexts. Therefore, this study mainly discusses the EEG components related to the expected stage and the feedback evaluation stage. The expected stage mainly focuses on cue-evoked P3 (Cue P3), and the feedback stage focuses on Feedback-evoked P3 (Fb-P3) and Feedback-evoked FRN (Fb-FRN) (Potts, 2011; White et al., 2021; Zhang et al., 2020).

### 2.5 Data analysis

The behavioral data and ERP data were analyzed separately using a 2(social context: SE/SI) × 2(reward category: MID/SID) × 2(reward magnitude: I/II) repeated-measures ANOVA. The Greenhouse–Geisser correction was used to compensate for sphericity violations. Least significant difference (LSD) was applied for *post-hoc* testing of main effects (Greimel et al., 2018). Partial eta-squared (η^2^p) has been reported as an indicator of the effect size in ANOVA tests. All of these statistical analyses were conducted using SPSS 25.0 software.

## 3 Results

### 3.1 Behavioral data: hit rate, reaction time

The results of repeated-measures ANOVA showed that the main effect of social context was significant (*F*_(1,26)_ = 8.115, *p* = 0.008, η^2^_p_ = 0.231), which showed that the hit rate of white squares under social inclusion was higher than that under social exclusion (Hit_SE_ vs. Hit_SI_ = 0.530 ± 0.006 vs. 0.541 ± 0.006). The main effects of reward category (*F*_(1,26)_ = 0.030, *p* = 0.864, η^2^_p_ = 0.001) and reward magnitude (*F*_(1,26)_ = 0.769, *p* = 0.388, η^2^_p_ = 0.028) were not significant, while ANOVA showed no significant interaction between the three variables.

The results of repeated-measures ANOVA at the time of reaction showed that the main effects of social context, reward category, and reward magnitude were all not significant. The repeated-measures ANOVA with pairwise interactions of the three variables also showed no significant interactions.

### 3.2 Event-related potentials

#### 3.2.1 Cue P3

**Figure 4:**
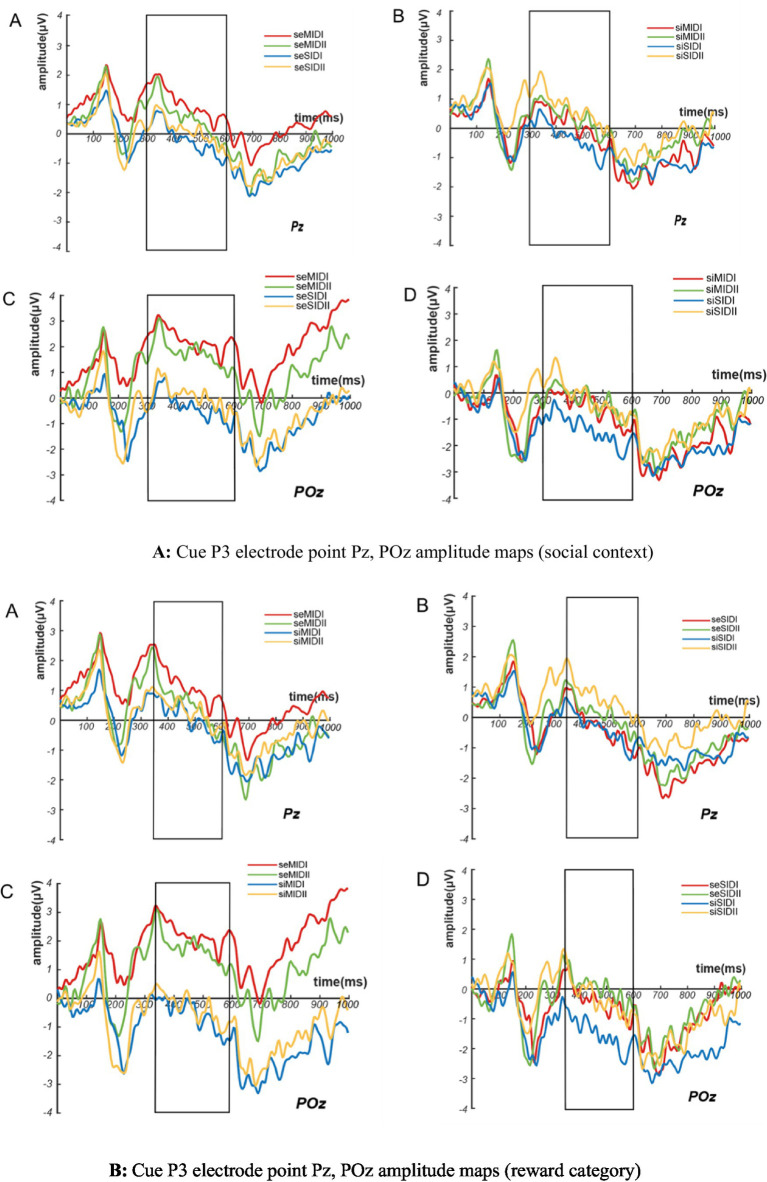
Amplitude maps of cue P3 electrode points Pz, POz for **(A)** social context and **(B)** reward category.

A 2(social context: SE/SI) × 2(reward category: MID/SID) × 2(reward magnitude: I/II) repeated-measures ANOVA on cued stimulus-induced Cue P3 wave amplitude found that the main effects of social context (*F*_(1,26)_ = 5.206, *p* = 0.031, η^2^_p_ = 0.167) and the reward category (*F*_(1,26)_ = 7.397, *p* = 0.011, η^2^_p_ = 0.221) were significant, but the main effect of reward magnitude was not significant. Cue P3 amplitude induced by cue stimulation under social exclusion was greater than that under social inclusion (SE vs. SI = 0.643 ± 0.672 *μV* vs. –0.182 ± 0.518 *μV*), and Cue P3 amplitude induced by cue stimulation under monetary reward conditions was greater than that under social reward conditions (MID vs. SID = 0.593 ± 0.651 *μV* vs. –0.131 ± 0.516 *μV*), as shown in Figures 4A,B. According to the results of Cue P3 amplitude stimulated by cues, deaf college students had stronger expectations and attention to social exclusion and monetary reward conditions than hearing college students.

**Figure 5:**
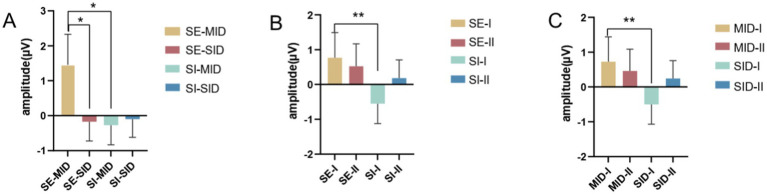
Results of the interaction analysis of three variables. **(A)** Interaction between social context and reward category. **(B)** Interaction between social context and reward magnitude. **(C)** Interaction between reward category and reward magnitude. Error bars represent the standard error of the mean. ^*^ *p* < 0.05.

The interaction analysis of the three variables showed that the interaction between social context and the reward category was significant (*F*_(1,26)_ = 5.464, *p* = 0.027, η^2^_p_ = 0.174), and under the conditions of social exclusion, the Cue P3 amplitude induced by monetary reward was greater than that induced by social reward (MID vs. SID = 1.452 ± 0.878 *μV* vs. –0.166 ± 0.548 *μV*). Similarly, under the conditions of social inclusion, the Cue P3 amplitude induced by monetary reward was greater than that induced by social reward (MID vs. SID = –0.266 ± 0.565 *μV* vs. –0.097 ± 0.516 *μV*). This shows that deaf college students have higher expectations of monetary reward cues under the conditions of both social situations. The interaction between social context and reward magnitude was significant (*F*_(1,26)_ = 5.504, *p* = 0.027, η^2^_p_ = 0.175), and under the conditions of social exclusion, Cue P3 amplitude caused by small reward cue stimulation was greater than that caused by large reward cue (small magnitude vs. large magnitude = 0.770 ± 0.722 *μV* vs. 0.516 ± 0.646 *μV*). Under the conditions of social inclusion, the Cue P3 amplitude induced by small reward cues was larger than that induced by large reward cues (small magnitude vs. large magnitude = −0.546 ± 0.566 μV vs. 0.182 ± 0.528 μV). The reward category and reward magnitude interaction was significant (*F*_(1,26)_ = 4.429, *p* = 0.045, η^2^_p_ = 0.146), and under the conditions of monetary reward, the Cue P3 amplitude caused by small reward cues was greater than that caused by large reward cues (small magnitude vs. large magnitude = 0.728 ± 0.711 *μV* vs. 0.457 ± 0.626 *μV*). Similarly, under the conditions of social reward, the Cue P3 amplitude caused by small reward cues was greater than that caused by large reward cues (small magnitude vs. large magnitude = −0.504 ± 0.566 *μV* vs. 0.242 ± 0.524 *μV*), see Figures 5A–C.

The interaction results further show that monetary reward cue stimuli are more likely to induce larger Cue P3 amplitudes than social reward stimuli. The difference is that small reward leads induce larger Cue P3 volatility than large reward leads. You will expect more from money but have a more conservative attitude toward how much you will eventually gain.

#### 3.2.2 Feedback-evoked P3 (Fb-P3)

**Figure 6:**
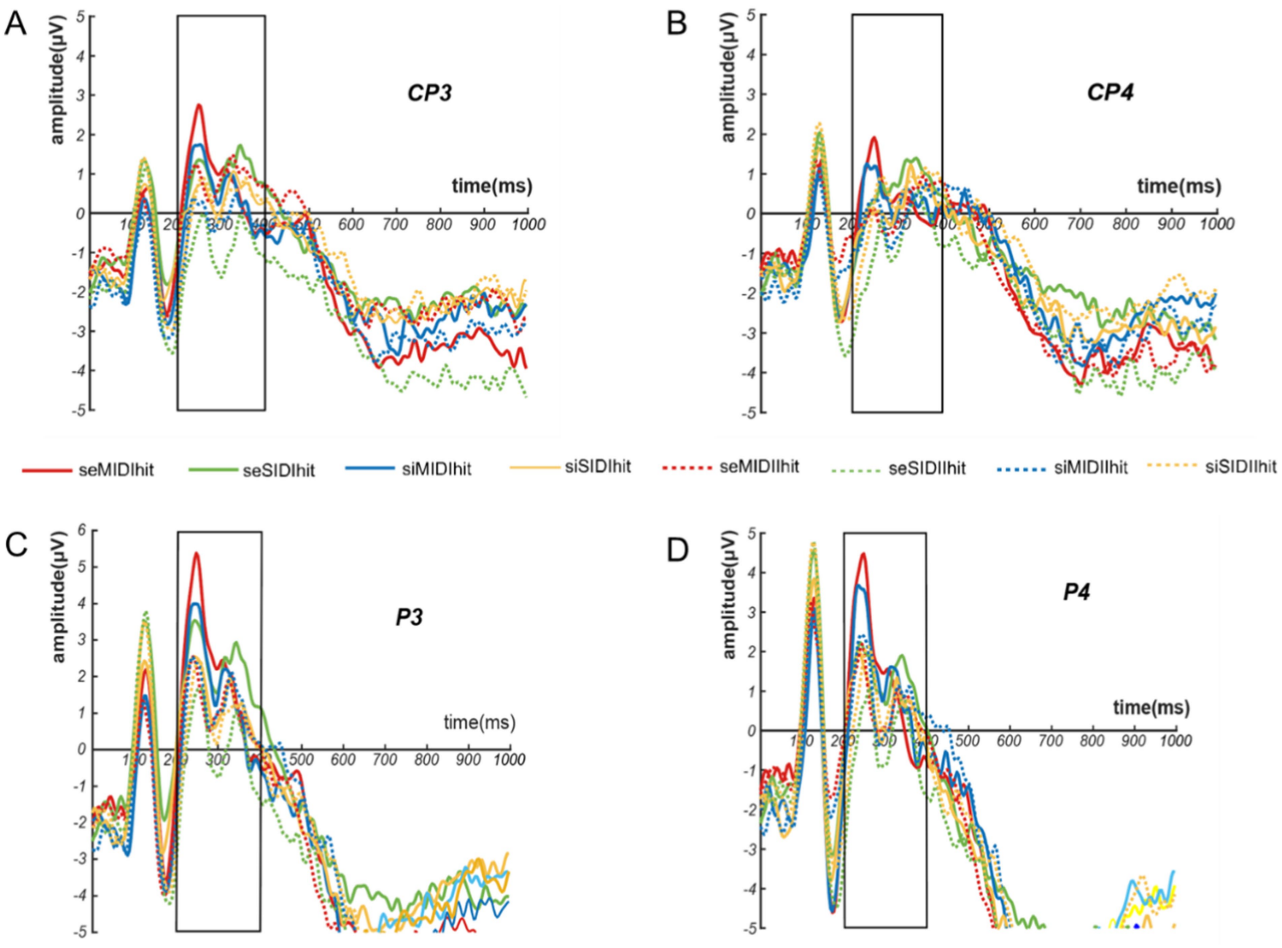
Amplitude maps of feedback-evokedP3 electrode points CP3**(A)**, CP4**(B)**, P3**(C)**, P4**(D)**.

Feedback-evoked P3 repeated-measures ANOVA with reward feedback showed that the main effect of reward magnitude was significant (*F*_(1,26)_ = 6.319, *p* = 0.018, η^2^_p_ = 0.196), and the Fb-P3 amplitude induced by small reward was larger than that induced by large reward (small magnitude vs. large magnitude = 0.939 ± 0.872 *μV* vs. 0.344 ± 857 *μV*), as shown in Figure 6. The main effects of social context and the reward category were not significant. The interaction between each of the two variables was not significant.

#### 3.2.3 Feedback-evoked FRN (Fb-FRN)

**Figure 7:**
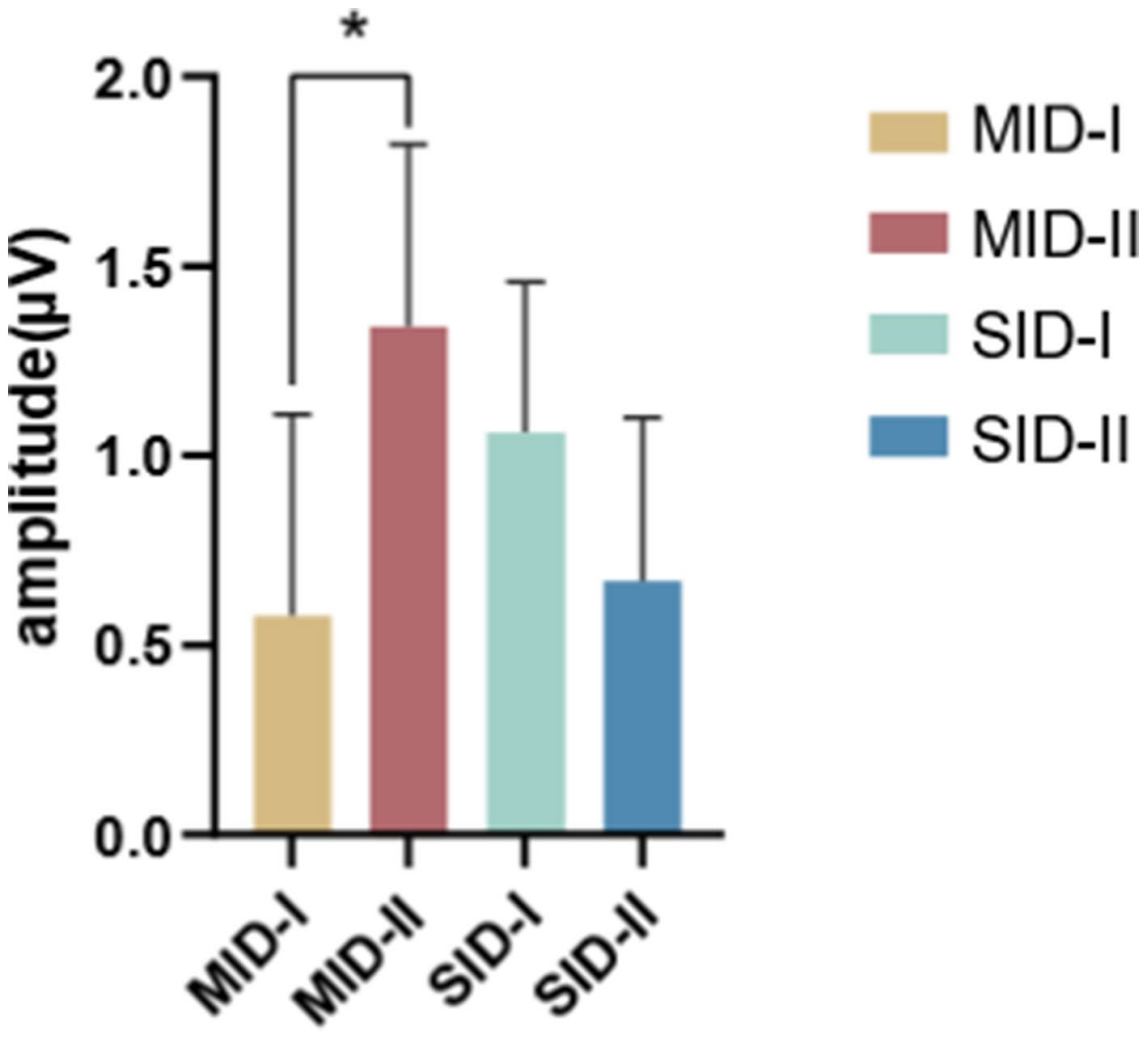
Interaction between the reward category and reward magnitude. Error bars represent the standard error of the mean. ^*^ *p* < 0.05.

A repeated-measures ANOVA of the FRN with rewards found that the main effects of social context, reward category, and reward magnitude were not significant. However, the reward category and reward magnitude interaction was significant (*F*_(1,26)_ = 8.57, *p* = 0.007, η^2^_p_ = 0.248), as evidenced by a larger magnitude with reward-evoked Fb-FRN amplitude under the monetary reward conditions (small magnitude vs. large magnitude = 0.578 ± 0.527 *μV* vs. 1.339 ± 0.484 *μV*), as shown in Figure 7.

## 4 Discussion

The present study explored the neurophysiological characteristics of reward processing in deaf college students under different social contexts with the MID and SID tasks by using ERP. At the behavioral level, there was a significant main effect of social context, such that the deaf college students showed a higher hit rate in the social inclusion context on the reward task. ERP results showed that reward cues evoked greater Cue P3 amplitude in the social exclusion context, and small-magnitude reward feedback evoked greater Feedback-evoked P3 amplitude than large-magnitude reward feedback. In addition, large-magnitude reward feedback elicited a larger FRN amplitude in the monetary reward cues than in the social reward cues.

Deaf college students exhibited a higher hit rate on the white square reward task in social inclusion. Furthermore, participants demonstrated a greater expectation of rewards and a higher hit rate when presented with social inclusion contextual pictures. One potential explanation is that people are born with an instinctive tendency and desire for social affiliation and interpersonal interaction (Ait Oumeziane et al., 2017; Baumeister and Leary, 1995). However, previous research has indicated that social exclusion may increase the expectation of monetary rewards and that negative experiences from exclusion may increase individuals’ pursuit of money (Feng et al., 2021; Lea and Webley, 2006). A review of the theory of pecuniary analgesia revealed that the experience of unpleasant pain caused by social exclusion motivates the pursuit of external relief, whereas the pleasurable sensations brought about by social inclusion may encourage the individual to a certain extent (Li et al., 2010). Consequently, the individual performed better in social inclusion, which is inconsistent with the ERP results possibly because behavioral outcomes can be affected by external factors that differ from EEG in time course. ERP technology has excellent temporal resolution, allowing comparison of the time course of social and monetary incentive processing (Ait Oumeziane et al., 2019; Greimel et al., 2018).

In the ERP results, the cue-evoked Cue P3 amplitude was greater in the social exclusion context than in the social inclusion context (Pfabigan et al., 2014). This indicated that social exclusion brought unpleasant experiences that strengthened individuals’ expectation and attention to rewards (Löw et al., 2008). Cue P3 is related to the allocation of attentional resources for decision-making or outcome evaluation, as well as motivation, reflecting the allocation of attention to reward-predicting cues (Ait Oumeziane et al., 2019; Ait Oumeziane et al., 2017), especially those stimuli that are task-relevant, infrequent, or unexpected (Courchesne et al., 1975; Donchin and Coles, 1988). Deaf college students may be too sensitive to specific information because of their hearing deficits and thus devote further attention resources (Bavelier et al., 2006; Marschark et al., 2013; Marschark et al., 2017). Previous studies have shown that social exclusion can lead to the development of unpleasant feelings such as internal isolation and self-doubt (Riva et al., 2016). To alleviate these feelings, individuals may attempt to mitigate the negative experience of social exclusion by monetarily enhancing their individual social charisma (Baumeister et al., 2008; Cristofori et al., 2015; Hess and Pickett, 2010; Li et al., 2010). Thus, deaf college students may have expectations and desires of monetary rewards following a series of situational pictures of social exclusion (Feng et al., 2021; Zhou et al., 2009). The greater Cue P3 amplitude elicited by the monetary reward cue in the social exclusion of the interaction also validated this idea (Lea and Webley, 2006). Moreover, the Cue P3 amplitude induced by the social exclusion context was greater in the small-magnitude reward cue condition than in the large-magnitude reward cue condition, which also suggested a greater effect of the social exclusion context (Xu et al., 2022). The results seemed to diverge from the prevailing view that reward expectation is proportional to reward magnitude (Dhingra et al., 2020) The greater the magnitude of the reward, the greater the activity in the brain regions activated by the reward expectation (Knutson et al., 2001), which suggested that a higher degree of expectation was associated with reward (Boukezzi et al., 2020). However, for the challenge of a new task, individuals may have minimal expectations, guaranteeing that they will be able to obtain the minimum gain before paying greater attention to and expecting larger expected rewards.

The P3 component is also related to the outcome of reward feedback (Gray et al., 2004; Leng and Zhou, 2010). The small magnitude of reward feedback induced a greater amplitude of Fb-P3, suggesting that deaf college students do pay attention to the small-rewarded task during the task and have a minimum expectation of reward acquisition (Ulrich et al., 2023; Wang et al., 2023). This indicated that deaf college students have conservative attitudes when facing task challenges (Batten et al., 2014; Fellinger et al., 2012). The minimum expectation mindset and the preference for monetary reward feedback respond to the complexity of expectations and feedback characteristics of deaf college students during the processing of rewards in different conditions, which reveals that for deaf college students there is a need for more practical and physical encouragement than hearing college students. Additionally, it is noteworthy that deaf college students in the monetary reward condition showed greater amplitude of the FRN in response to substantial reward feedback. This indicated that deaf college students are more concerned about obtaining greater monetary gains (Feng et al., 2021; Jia et al., 2011), exhibiting a heightened sense of favoritism toward monetary rewards (Altikulaç et al., 2019; Rademacher et al., 2014) and a more pronounced emotional involvement (Yuan et al., 2012). Despite a greater concern for smaller reward cues, this did not conflict with the desire to gain more, suggesting that deaf college students are pragmatic in their approach to tasks and benefits.

There are some limitations to this study, and potential future directions should be noted. First, the sample size of this study was small and the study group was single with only deaf college students; therefore, the findings may not be generalizable to other deaf groups. Future research could investigate the causes of developmental delays in deaf individuals by increasing the number of subjects and subject groups, as well as considering variables that may be associated with reward processing. These variables could include the degree of hearing loss, academic environment, socialization, and cognitive experiences. Second, the study did not adequately take into account that the deaf participants may have different interpretations, emotional experiences, and neural responses to context-based social images when selecting stimuli and can be further explored in future studies for the emotional attributes of facial expressions and social context to balance experimental control with ecological validity. Furthermore, the current study lacked a hearing control group, and a multifactorial mixed design could be used in the future to include a control group for comparative studies. Finally, the category of rewards in this study only examined money and social context. A subsequent study should investigate the rewards that deaf college students value or dislike to develop a deeper understanding of their reward characteristics.

## 5 Conclusion

In conclusion, deaf college students were more concerned with monetary rewards in the context of social exclusion. Additionally, their concern for small reward cues and feedback suggests that they approached the tasks in this study with a minimum expectation mentality.

## Data Availability

The raw data supporting the conclusions of this article will be made available by the authors, without undue reservation.
